# Retrospective analysis on the clinical outcomes of recombinant human soluble thrombomodulin for disseminated intravascular coagulation syndrome associated with solid tumors

**DOI:** 10.1007/s10147-018-1261-z

**Published:** 2018-03-07

**Authors:** Kota Ouchi, Shin Takahashi, Sonoko Chikamatsu, Shukuei Ito, Yoshikazu Takahashi, Sadayuki Kawai, Akira Okita, Yuki Kasahara, Yoshinari Okada, Hiroo Imai, Keigo Komine, Ken Saijo, Masahiro Takahashi, Hidekazu Shirota, Masanobu Takahashi, Makio Gamoh, Chikashi Ishioka

**Affiliations:** 1 Department of Medical Oncology, Tohoku University Hospital, Sendai, Japan; 2 Department of Clinical Oncology, Institute of Development, Aging and Cancer, Tohoku University, 4-1, Seiryo-machi, Aobaku Sendai, 980-8575 Japan; 3 Department of Medical Oncology, Osaki Citizen Hospital, Osaki, Japan

**Keywords:** Disseminated intravascular coagulation, Recombinant human soluble thrombomodulin, Solid tumors

## Abstract

**Background:**

Recombinant human soluble thrombomodulin (rTM) has been established and introduced in the clinic as a standard treatment for disseminated intravascular coagulation (DIC). However, the efficacy and safety of rTM for DIC associated with solid tumors (DIC-STs) have not been fully established. Here, we performed a retrospective analysis of the clinical outcomes of rTM for DIC-STs and considered a treatment strategy with rTM for DIC-STs.

**Methods:**

Patients with DIC-STs between November 2009 and March 2016 in 2 cancer core hospitals were retrospectively analyzed. Data, including patient background, treatment course, and clinical outcomes of rTM for DIC-STs, were extracted. The clinical outcomes were evaluated by comparing the DIC score, resolution rate, and overall survival (OS) duration.

**Results:**

The study included 123 patients with DIC-STs. The median OS in all patients was 41 days. The DIC resolution rate was 35.2%. DIC scores and DIC-related blood test data (fibrin degradation product and prothrombin time-international normalized ratio) significantly improved at the end of rTM administration (*P* < 0.001). The OS duration was longer in patients who were treated with chemotherapy after DIC onset than in those who were not treated with chemotherapy (median, 178 days vs. 17 days, *P* < 0.001). In both univariate and multivariate analyses, chemotherapy after DIC onset showed the strongest association with OS.

**Conclusions:**

rTM can at least temporarily improve or maintain the state of DIC-STs. It is suggested that prolongation of survival can be expected when control of DIC and treatment of the underlying disease are compatible.

**Electronic supplementary material:**

The online version of this article (10.1007/s10147-018-1261-z) contains supplementary material, which is available to authorized users.

## Introduction

Disseminated intravascular coagulation (DIC) is a disease state in which microscopic thrombi frequently occur in microvessels, resulting in remarkable coagulation activation with systemic persistence in the presence of an underlying disease [1]. In patients with solid tumors, the extrinsic coagulation mechanism involving tissue factors on the tumor cell surface and in tumor cells is mainly activated [2]. Therefore, along with acute leukemia and sepsis, this is extremely important as an underlying condition of DIC [3].

With regard to the treatment strategy for DIC, treatment of the underlying disease is the most effective approach [4]; however, supportive therapy to prevent rapid exacerbation of coagulopathy has also been used in combination. Although heparin is considered to interfere with over-activation of the coagulation system in DIC, no randomized-controlled trial has demonstrated that the prognosis of DIC patients significantly improved with its administration [5, 6]. On the other hand, recombinant human soluble thrombomodulin (thrombomodulin alfa, rTM) has been shown to be effective against DIC triggered by a hematologic malignancy and sepsis [7].

The prognosis of metastatic solid tumor patients who develop DIC has been reported to be extremely poor [8]. Recently, the results of a prospective study examining the effectiveness of rTM for DIC associated with solid tumors (DIC-STs) have been reported [9]. In this previous study, it was shown that rTM was effective and could improve DIC in cases of DIC-STs. However, the follow-up period was limited to 28 days; thus, long-term survival data were not presented. In addition, only patients with a life expectancy of over 28 days after recovery from DIC were included in that study [9]. Thus, the clinical outcomes of rTM for DIC-STs in clinical practice are not clear. Moreover, a treatment strategy for DIC-STs has not been established.

Therefore, the present retrospective analysis aimed to examine the clinical outcomes of rTM for DIC-STs in clinical practice and consider a treatment strategy with rTM for DIC-STs.

## Patients and methods

### Patients

The present study included patients who received rTM for diagnosed/suspected DIC-STs between November 2009 and March 2016, at Tohoku University Hospital and Osaki Citizen Hospital.

### Patient extraction and diagnostic criteria

According to medical records between November 2009 and February 2016, patients with metastatic solid tumors who developed DIC or were suspected to have DIC and who were administered rTM at Tohoku University Hospital and Osaki Citizen Hospital were identified. DIC was diagnosed according to the Japanese Ministry of Health and Welfare (JMHW) DIC criteria [10]. The DIC score was calculated using medical record descriptions and blood test data just before rTM administration. Patients who underwent chemotherapy within 21 days before rTM administration and who showed a reduction in the number of erythrocytes or white blood cells after chemotherapy compared with before chemotherapy were included in the myelosuppressive group. Patients who did not show myelosuppression were included in the non-myelosuppressive group. In the non-myelosuppressive group, DIC was diagnosed when a DIC score of 7 or more was calculated and DIC was suspected when a score of 6 was calculated. In the myelosuppressive group, the scores of platelets and bleeding symptoms were both 0, and DIC was diagnosed or suspected if the total score of other factors was 3 or more. rTM was administered intravenously once daily at 380 U/kg over 30 min. In patients, rTM was administered at least once, and the subsequent administration period was decided by the doctor according to the reaction to the treatment and the condition of the patient.

### Performance status at DIC onset

The performance status (PS) at DIC onset was determined according to the Eastern Cooperative Oncology Group (ECOG) Performance Status.

### Cause of DIC

The cause of DIC was classified as “tumor,” “infection,” or “indistinguishable.” When blood culture at DIC onset was positive, an infected focus was identified through imaging (radiography, computed tomography, or magnetic resonance imaging), and/or clinically obvious infection was noted, DIC was considered to be caused by infection. When tumor marker elevation was noted and/or tumor progression was identified on imaging, without infectious findings, DIC was considered to be caused by a tumor. When both infectious findings and tumor progression were confirmed, the cause of DIC was considered indistinguishable.

### Evaluation of clinical outcomes

The clinical outcomes of rTM were evaluated by comparing DIC scores and related blood test data between before and at the end of rTM administration, according to the presence or absence of DIC resolution and the overall survival (OS) duration. For the DIC scores and related blood test data [fibrin degradation product (FDP), platelet count, prothrombin time-international normalized ratio (PT-INR), and fibrinogen], the results immediately before rTM administration were compared with the results at the end of rTM administration. If examination was not conducted at the end of rTM administration, evaluation was performed using the latest test results after administration. DIC resolution was judged according to the DIC score. OS duration was defined as the period from the start of rTM administration to patient death. Survival cases at the end of the observation period were censored.

### Clinical outcomes of conventional treatment agents for DIC-STs

Patients who received other anticoagulant agents (gabexate mesilate and/or dalteparin sodium) for DIC-STs between August 2007 and February 2010 at Tohoku University Hospital were included in a conventional treatment cohort. The clinical outcomes (OS duration and DIC resolution rate) of this cohort were compared with the outcomes of the rTM cohort.

### Statistical analysis

Statistical analyses of case backgrounds and clinical courses were conducted using JMP Pro 13 (SAS Institute Inc., Cary, NC, USA). In comparisons of case backgrounds, Chi-square tests were performed to assess significance. Differences in DIC-related test results between before and at the end of rTM administration were statistically assessed using the Wilcoxon rank-sum test. Survival curves were prepared using the Kaplan–Meier method, and differences were assessed using the log-rank method. Associations between patient factors, including chemotherapy after DIC onset and OS after rTM administration, were used in univariate and multivariate analyses with a Cox proportion hazard model. A *P* < 0.05 was considered to indicate statistical significance.

## Results

### Baseline characteristics of the study patients

A total of 139 patients with metastatic solid tumors who received rTM for DIC were considered for inclusion (75 and 64 patients were treated at Tohoku University Hospital and Osaki Citizen Hospital, respectively). However, 16 patients were excluded, because they did not meet the requirement for suspected DIC or DIC according to the JMHW DIC criteria. Therefore, 123 patients were analyzed. The median follow-up period was 29 days (range 1–957 days). Of these 123 patients, 15 died shortly after the start of rTM administration, and DIC-related test data at the end of treatment could not be obtained. Therefore, 108 patients were included in the analysis of the DIC resolution rate and in the comparison of DIC-related test data. The backgrounds of the 123 patients diagnosed with DIC or suspected with DIC are shown in Table 1. As the patients had advanced solid tumors with distant metastasis, more than half of the included patients had PS 3 or 4 (PS 3, 37.4%; PS 4, 22.0%). In addition, approximately half of the patients (52.8%) received 2 or more chemotherapeutic regimens at DIC onset. On the other hand, some included patients (13.0%) had not received chemotherapy. The proportion of DIC caused by infection was the highest (51.2%), followed by DIC caused by tumors (38.2%). With regard to the types of tumors as underlying diseases, gastric cancer (31.7%), colon cancer (20.3%), pancreatic cancer (9.8%), biliary tract cancer (8.1%), and esophageal cancer (7.3%) were frequently observed (Table 1).

**Table 1:** Baseline characteristics of the patients

	*n*	(%)
Sex		
Male	87	(70.7)
Female	36	(29.3)
Median age (range)	65 (range 30–84)	
Performance status		
0	1	(0.8)
1	8	(6.5)
2	41	(33.3)
3	46	(37.4)
4	27	(22.0)
Number of previous regimens		
0	16	(13.0)
1	42	(34.2)
2	40	(32.5)
≥ 3	25	(20.3)
Cause of DIC		
Tumor^a^	47	(38.2)
Infection^b^	63	(51.2)
Indistinguishable^c^	13	(10.6)
Bone marrow suppression		
+	38	(30.9)
−	85	(69.1)
Types of tumors		
Gastric cancer	39	(31.7)
Colorectal cancer	25	(20.3)
Pancreatic cancer	12	(9.8)
Biliary tract cancer	10	(8.1)
Esophageal cancer	9	(7.3)
Cancer of unknown primary origin	4	(3.3)
Lung cancer	4	(3.3)
Gastrointestinal stromal tumor	3	(2.4)
Ewing sarcoma	2	(1.6)
Breast cancer	2	(1.6)
Neuroendocrine tumor	2	(1.6)
Others	11	(8.9)

^a^Tumor: Cases with cancer progression without definite infection findings

^b^Infection: Cases with clinically obvious infection and/or blood culture positivity

^c^Indistinguishable: Cases with both cancer progression and definite infection findings

### Clinical outcomes

#### Overall survival, DIC resolution rate, and DIC-related blood test data

**Fig. 1:**
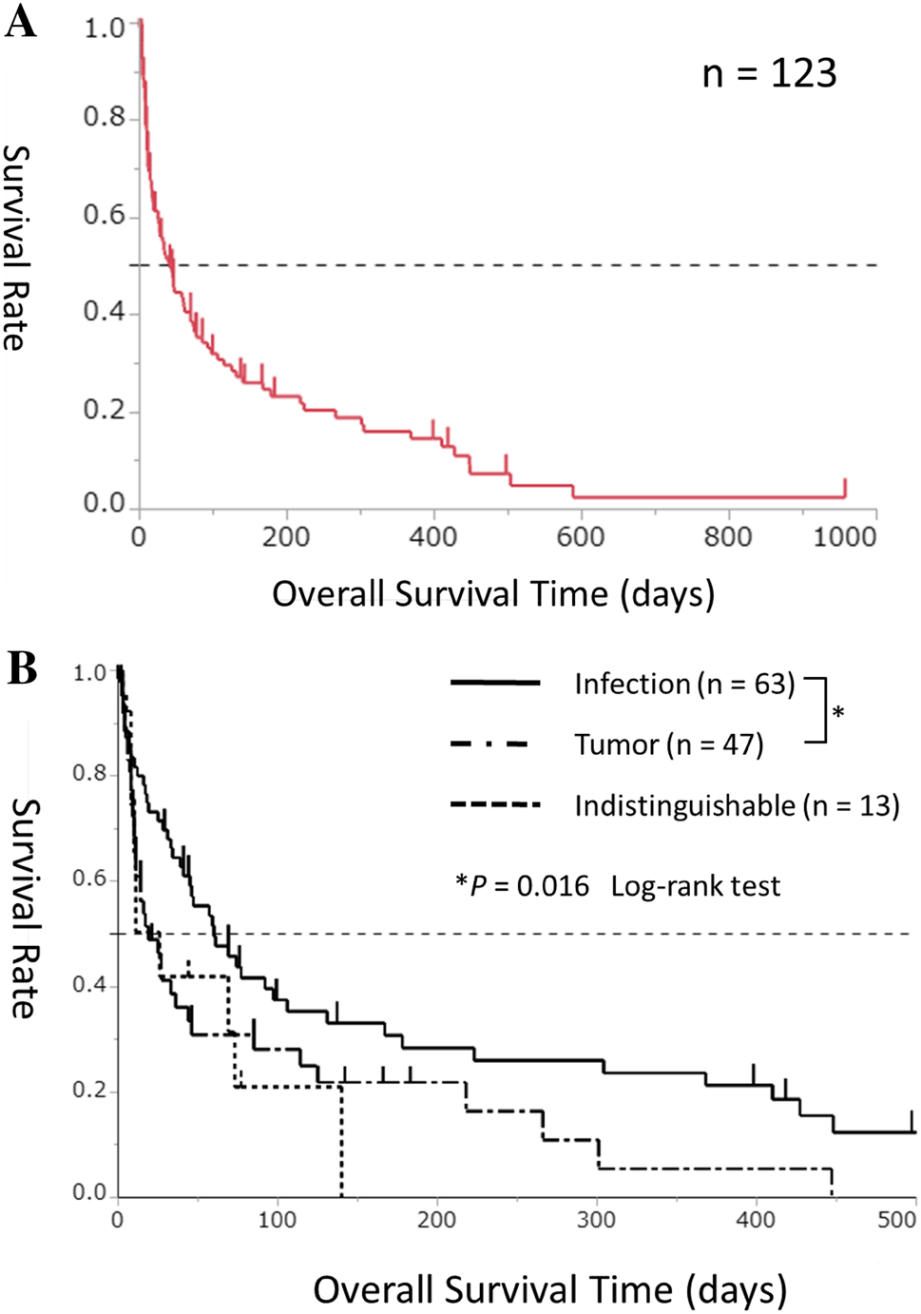
**a** Kaplan–Meier survival curves for overall survival duration in the 123 study patients. **b** Kaplan–Meier survival curves for overall survival duration in the infection-associated group (solid line, *n* = 63), tumor-associated group (chain line, *n = *47), and indistinguishable group (dotted line, *n* = 13)

The median OS in all patients (*n* = 123) was 41 days (range 1–957), and the survival rate at 28 days was 52.0% (Fig. 1a). All deaths were caused by progression of the tumor or infection, and no death was associated with severe side effects caused by rTM administration.

**Fig. 2:**
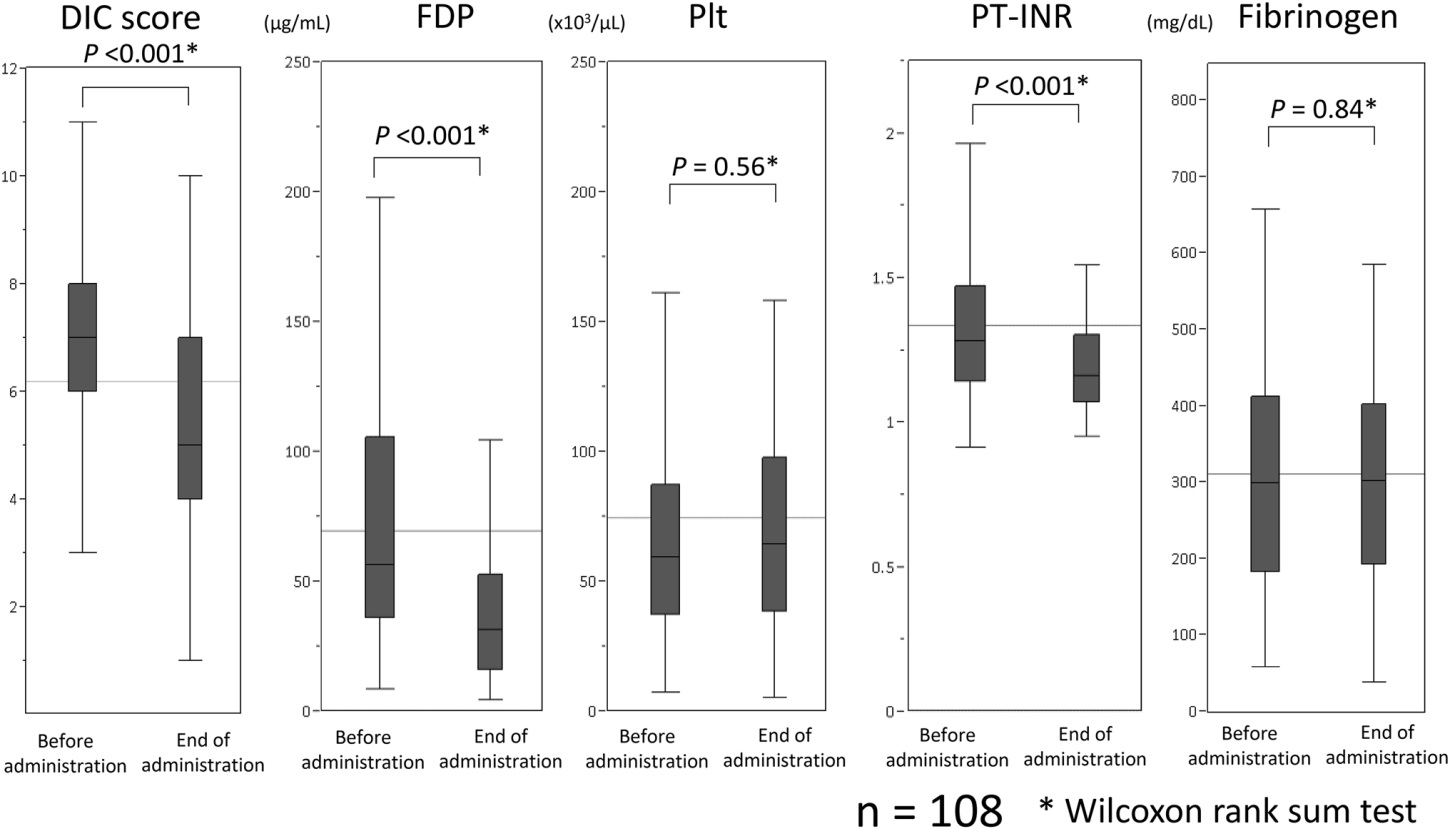
Comparison of disseminated intravascular coagulation scores and related data between before and at the end of recombinant human soluble thrombomodulin administration in 108 patients

Comparison of DIC scores in the 108 patients who underwent DIC-related tests both before and at the end of rTM administration showed a significant improvement in the DIC score at the end of rTM administration when compared with the score before administration (*P*<0.001, Fig. 2). Comparison of DIC-related blood test data showed a significant improvement in FDP and PT-INR at the end of rTM administration (*P*<0.001 and *P*<0.001, respectively); however, there was no significant difference with regard to platelet count and fibrinogen level (*P* = 0.56 and *P* = 0.84, respectively, Fig. 2).

The DIC resolution rates were 35.2% among the 108 patients, 29.3% in the tumor-associated group, and 44.8% in the infection-associated group (*P*=0.11, Table 2).

**Table 2:** Comparison of patient background according to DIC resolution

		Total	Resolved	Not resolved	Resolution rate (%)	*P* value^a^
			(*n* = 38)	(*n* = 70)		
Total		108	38	70	35.2	
Age						
≥ 65 years		55	20	35	36.4	0.79
≤ 64 years		53	18	35	34.0	
Performance status						
≤ 2		45	18	27	40.0	0.38
≥ 3		63	20	43	31.7	
Number of previous regimens						
≤ 2		88	29	59	33.0	0.31
≥ 3		20	9	11	45.0	
Cause of DIC						
Tumor		41	12	29	29.3	0.11^b^
Infection		58	26	32	44.8	
Indistinguishable		9	0	9	0.0	

*DIC* disseminated intravascular coagulation

^a^*X*^2^ test

^b^Tumor vs. infection

**Fig. 3:**
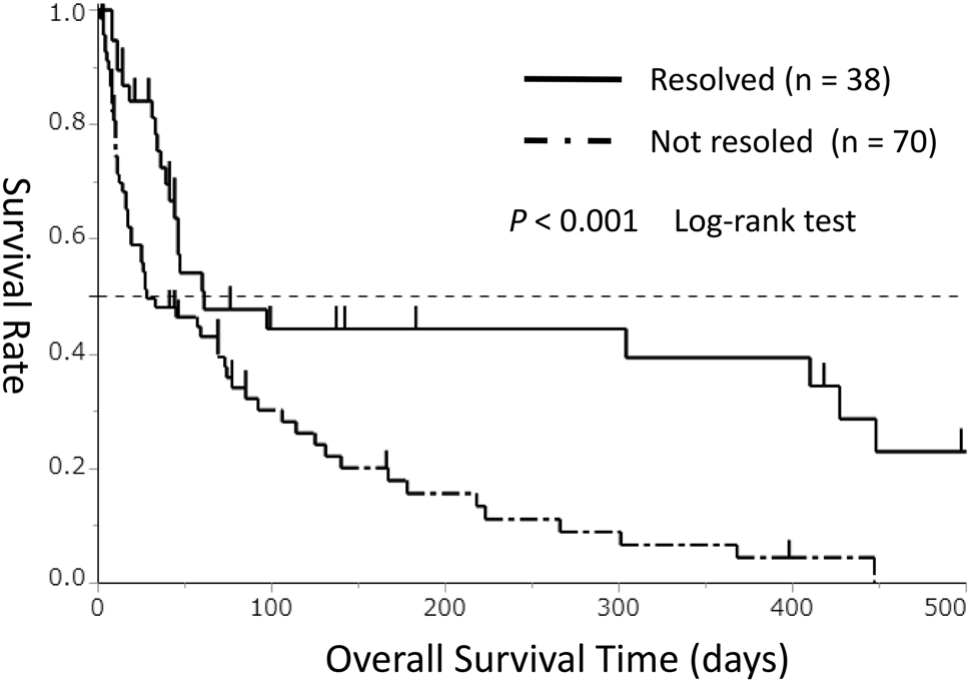
Kaplan–Meier survival curves for overall survival duration in the disseminated intravascular coagulation resolved group (solid line, *n* = 38) and non-resolved group (chain line, *n* = 70)

The 108 patients were divided into 2 groups according to the presence (*n* = 38) or absence (*n* = 70) of DIC resolution, and OS was compared between the groups. The OS duration was significantly longer in the DIC resolved group than in the DIC non-resolved group (median, 61 vs. 28 days, *P*<0.001, Fig. 3).

#### Comparison of clinical outcomes between rTM and conventional treatment agents for DIC-STs

To compare the clinical outcomes of rTM for DIC-STs with the outcomes of conventional treatment, we retrospectively assessed 19 patients who received other anticoagulant agents (gabexate mesilate and/or dalteparin sodium). The background of these patients is presented in Online Resource 1. The OS duration was significantly longer in the rTM cohort than in the conventional treatment cohort (median, 41 vs. 14 days, *P*=0.002, Online Resource 2), and the DIC resolution rate tended to be higher in the rTM cohort than in the conventional treatment cohort, although the difference was not significant (35.2 vs. 15.8%, *P*=0.08, Online Resource 3).

#### Comparison of patient background according to the presence or absence of DIC resolution

To identify the factors that affected DIC resolution, we compared patient background between the DIC resolved group and DIC non-resolved group. There were no significant differences in terms of age, PS, the number of previous regimens, and the cause of DIC between the groups (Table 2).

#### Comparison of survival time according to the cause of DIC

On comparing OS according to the cause of DIC, a significantly longer OS duration was observed in the infection-associated group (*n* = 63) than in the tumor-associated group (*n* = 47) (median, 60 vs. 19 days, *P*=0.016, Fig. 1b).

#### Comparison of survival duration according to the presence or absence of chemotherapy after DIC onset

**Fig. 4:**
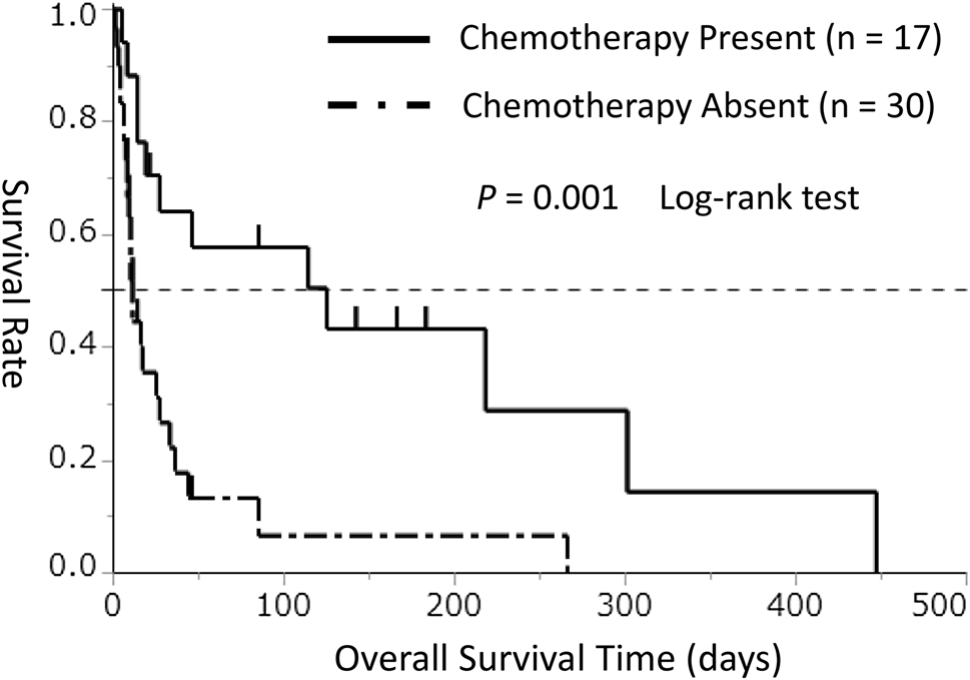
Kaplan–Meier survival curves for overall survival duration in the chemotherapy group (solid line) and no chemotherapy group (chain line) among patients with tumor-associated disseminated intravascular coagulation (*n* = 47)

Treatment of the underlying disease is considered to be extremely important in the treatment of DIC [4]. All DIC cases associated with infection were receiving treatment for the underlying disease. On the other hand, among DIC cases associated with tumors, only 36% (17/47 cases) were treated with anticancer drugs after DIC onset. Thus, to clarify the relationship between the treatment of the underlying disease and OS, we analyzed OS according to the presence or absence of chemotherapy after DIC onset in the tumor-associated group (*n* = 47). In this analysis, the OS duration was significantly longer in the chemotherapy group (*n* = 17) than in the no chemotherapy group (*n* = 30) (median, 125 vs. 11 days, *P*=0.001, Fig. 4).

To investigate the factors related to the presence or absence of chemotherapy after DIC onset in the clinical setting, we compared patient background (age, PS, number of previous regimens, and DIC resolution) between subgroups. The proportion of cases of PS 0–2 was higher in the chemotherapy group than in the no chemotherapy group (*P*=0.002). The number of previous regimens tended to be lower in the chemotherapy group than in the no chemotherapy group (*P*=0.06, Table 3).

**Table 3:** Comparison of patient background according to chemotherapy

	Total	Chemotherapy		*P* value^a^
		Present (*n* = 17)	Absent (*n* = 30)	
Age				
≥ 65 years	18	6	12	0.75
≤ 64 years	29	11	18	
Performance status				
≤ 2	25	14	11	0.002
≥ 3	22	3	19	
Number of previous regimens				
≤ 2	38	16	22	0.06
≥ 3	9	1	8	
Resolution of DIC				
Resolved	12	6	6	0.48
Not resolved	29	11	18	
NA	6	0	6	

*DIC* disseminated intravascular coagulation

^a^*X*^2^ test

### Univariate and multivariate analyses for OS

To identify the factors associated with OS, 6 parameters (age, number of previous regimens, chemotherapy after DIC onset, PS, cause of DIC, and resolution of DIC) were evaluated using Cox regression analyses. In both univariate and multivariate analyses, chemotherapy after DIC onset showed the strongest association with OS (HR=0.25; 95% CI, 0.15–0.43; *P* < 0.001 and HR=0.34; 95% CI, 0.18–0.64; *P* = 0.001, respectively; Table 4).

**Table 4:** Cox regression analysis for clinical outcomes of rTM

	*n*	Univariate			Multivariate		
		HR	95% CI	*P* value^a^	HR	95% CI	*P* value^a^
Age							
≥ 65 years	50	0.74	(0.46–1.17)	0.20			
≤ 64 years	49						
Number of previous regimens							
≤ 2	80	1	(0.57–1.88)	0.80			
≥ 3	19						
Chemotherapy after onset of DIC							
Present	40	0.25	(0.15–0.43)	< 0.001	0.34	(0.18–0.64)	0.001
Absent	59						
Performance status							
≤ 2	42	0.42	(0.25–0.68)	0.001	0.60	(0.33–1.80)	0.09
≥ 3	57						
Cause of DIC							
Tumor	41	1.76	(1.09–2.82)	0.02	1.67	(1.01–2.75)	0.05
Infection	58						
Resolution of DIC							
Resolved	38	0.43	(0.25–0.71)	0.001	0.52	(0.30–0.88)	0.01
Not resolved	61						

*rTM* recombinant thrombomodulin, *DIC* disseminated intravascular coagulation

^a^*X*^2^ test

## Discussion

To our knowledge, this is the first report on the long-term follow-up of more than 100 cases to assess the clinical outcomes of rTM for DIC-STs. In addition, we focused on the treatment of the underlying disease of DIC. This approach is unique and differs from the approach in a previous report [9].

About half of the cases received 0 or 1 previous chemotherapeutic regimen. This finding suggests that there is a high possibility of DIC occurrence from the early phase of the clinical course, and this is not limited to patients with a long treatment history. It is known that the risk of DIC complication is high in cases with large tumors and distant metastases [11], and in such cases, periodic monitoring of blood coagulation-related factors has been considered useful for the early detection and treatment of DIC. In our study, among the DIC cases associated with solid tumors, the cause of DIC was considered as infection in approximately half of the cases (51.2%). As shown in a previous study [7], DIC cases associated with infectious diseases showed good outcomes and were considered to be appropriate for positive treatment using antibiotics. Thus, it is important to diagnose the cause of DIC through blood culture or image inspection.

As shown in a previous study by Tamura et al. [9], rTM administration can improve or maintain blood coagulation-related data associated with the DIC score and DIC diagnostic criteria. One of the possible reasons for the absence of a difference in the platelet count was chemotherapy-mediated bone marrow suppression. In such cases, recovery of the platelet count would be difficult even if the abnormality of the coagulation system shows improvement. The absence of a significant difference in the fibrinogen level was considered to be associated with the fact that cases with a decreased fibrinogen level, which has an influence on score calculation, were rare at the time of DIC diagnosis. A previous study showed that fibrinogen levels increase when a solid tumor is the underlying disease [12]; thus, the fibrinogen level hardly decreases with increased consumption of fibrinogen. OS results indicated that DIC resolution contributed to an improved prognosis. Although these findings have been reported in a previous study [9], the present study showed the significance of rTM use in clinical practice.

In a previous report, 8 patients who developed DIC-STs were treated with heparin, fresh frozen plasma, and platelet support, but only 1 patient showed short-term improvement in the clinical condition and coagulation test results [13]. On the other hand, in the prospective study by Tamura et al., 10 of 25 patients with DIC-STs, who did not receive treatment for the underlying disease (antineoplastic agents), showed improvement in the DIC score after rTM administration, and 7 patients experienced recovery from DIC [9]. On comparing the clinical outcomes between rTM and conventional treatment, it was found that the outcomes of rTM were better. However, the sample size in the conventional treatment group was small, and there might have been various biases (e.g., the chemotherapy regimens that could be selected differed as the time of treatment differed). Thus, the result is only exploratory, and it is not intended to prove the superiority of rTM over the conventional anticoagulant agents.

The clinical outcomes of rTM for DIC-STs were equivalent to those reported previously for the entire patient group (Online Resource 4) [9, 14]. Even in actual clinical practice, it was found that 1 of every 3 cases could be expected to achieve DIC resolution.

In the analysis according to the cause of DIC, there was no significant difference in the resolution rate. However, in the comparison of OS, the survival duration tended to be shorter in patients who developed DIC because of tumor progression than in patients who developed DIC because of infection. This result is thought to be associated with differences in the treatment rates of underlying diseases, which are considered to be extremely important in DIC treatment. It was demonstrated that treatment of the underlying disease (chemotherapy) strongly influences the survival time, even in cases of DIC associated with tumor progression. Some reports have suggested that prognosis may be prolonged with chemotherapy for DIC-STs [15, 16]. On the other hand, in the presence of DIC, it is difficult to carry out chemotherapy because of its toxicity and invasiveness. This is a dilemma with regard to the treatment strategy for DIC-STs. Therefore, control of DIC with rTM could significantly support the adoption of chemotherapy in patients with DIC-STs. In patients with DIC caused by tumors, rTM treatment could provide an opportunity for the use of chemotherapy in the treatment of the underlying disease.

We found that PS at DIC onset was significantly associated with chemotherapy administration. Thus, PS can be considered as a good criterion to judge whether chemotherapy can be performed. The proportion of cases with 2 or less previous regimens tended to be higher among cases that received chemotherapy than among those that did not receive chemotherapy, although the difference was not significant. In patients who have not used standard chemotherapy regimens, chemotherapy can be administered if DIC control can be achieved. Based on these results, a treatment strategy involving the active control of DIC with rTM and subsequent chemotherapy can be effective in patients with a relatively good PS (2 or less) and those who have not used standard chemotherapy regimens. Chemotherapy after DIC onset was an independent factor and was strongly associated with OS (Table 4). Thus, it is important to consider chemotherapy administration for the improvement of OS in patients with DIC-STs, regardless of the cause of DIC.

New drugs, such as rTM, are often more expensive than the conventional agents. To our knowledge, there has been no report on the cost effectiveness of rTM for DIC-STs, and we do not have data to discuss the cost effectiveness. However, cost effectiveness can be improved by administering rTM only to patients who can be treated for the underlying disease of DIC rather than administering it to all patients with DIC-STs.

As this was a retrospective study, there were wide variations in the baseline characteristics of the patients, combination therapies, and examination data at the time of DIC onset. Furthermore, there might have been some selection bias before rTM treatment choice. On the other hand, it can be considered that the study was carried out with a patient group in actual clinical practice. The number of cases in this study was too small for subgroup analysis; thus, it is necessary to further accumulate cases in the future and verify our results. In addition, the clinical outcomes in this study were influenced by combination drugs that were mainly administered for the treatment of the underlying disease; thus, it was difficult to thoroughly evaluate the effectiveness of rTM. Although a prospective study by Tamura et al. [9] reported the efficacy of rTM, to strictly evaluate the effectiveness of rTM, a prospective study comparing the results between a treatment group and a control group is desired in the future.

In conclusion, the DIC score and related coagulation data can be temporarily controlled with rTM administration in cases of DIC-STs. In addition, treatment of the underlying disease is important in the treatment of DIC. Our results suggested that prolongation of survival can be expected when control of DIC and treatment of the underlying disease are compatible. Especially, in patients with DIC caused by tumors, rTM treatment could allow adoption of chemotherapy for the underlying disease of DIC.

## Electronic supplementary material

Below is the link to the electronic supplementary material.

Supplementary material 1 (DOCX 146 kb)
