# Supplementary material for: Retrospective analysis on the clinical outcomes of recombinant human soluble thrombomodulin for disseminated intravascular coagulation syndrome associated with solid tumors
Source: Int J Clin Oncol. 2018 Mar 7;23(4):790–8. doi: 10.1007/s10147-018-1261-z (PMC6097084; doi:10.1007/s10147-018-1261-z)
Supplement: Supplementary file 1 — Supplementary material 1 (DOCX 146 kb) [file 10147_2018_1261_MOESM1_ESM.docx]

Online Resource

**Article Title:**

Retrospective Analysis on the Clinical Outcomes of Recombinant Human Soluble Thrombomodulin for Disseminated Intravascular Coagulation Syndrome associated with Solid Tumors

**Journal Name:**

*International Journal of Clinical Oncology*

**Authors**:

Kota Ouchi^1,2^, Shin takahashi^1,2^, Sonoko Chikamatsu^1,2^, Shukuei Ito^1,2^, Yoshikazu Takahashi^3^, Sadayuki Kawai^3^, Akira Okita^1,2^, Yuki Kasahara^1,2^, Yoshinari Okada^1,2^, Hiroo Imai^1,2^, Keigo Komine^1,2^, Ken Saijo^1,2^, Masahiro Takahashi^1,2^, Hidekazu Shirota^1,2^, Masanobu Takahashi^1,2^, Makio Gamoh^3^, and Chikashi Ishioka^1,2^

**Affiliation**:

1. Department of Medical Oncology, Tohoku University Hospital
2. Department of Clinical Oncology, Institute of Development, Aging and Cancer, Tohoku University
3. Department of Medical Oncology, Osaki Citizen Hospital

**Corresponding Author**: Chikashi Ishioka

**E-mail**: [chikashi@tohoku.ac.jp](mailto:chikashi@tohoku.ac.jp)

*Online Resource 1.* Baseline characteristics of patients who received conventional treatment *…...............……….……………………...…………………………………………………….* 3

*Online Resource 2.* Kaplan–Meier survival curves for overall survival duration in the rTM cohort and conventional treatment cohort …………………………………………………. 4

Figure legend of Online Resource 2. ……………………………………………………..... 4

*Online Resource 3.* The resolution rate from DIC in the rTM cohort and conventional treatment cohort*.....…………………………………………………………………………...* 5

*Online Resource 4.* Retrospective/prospective study on the efficacy of rTM for DIC complicated with solid tumors *…..……………………………………………………….…..* 6

Online Resource 1

Online Resource 2

*
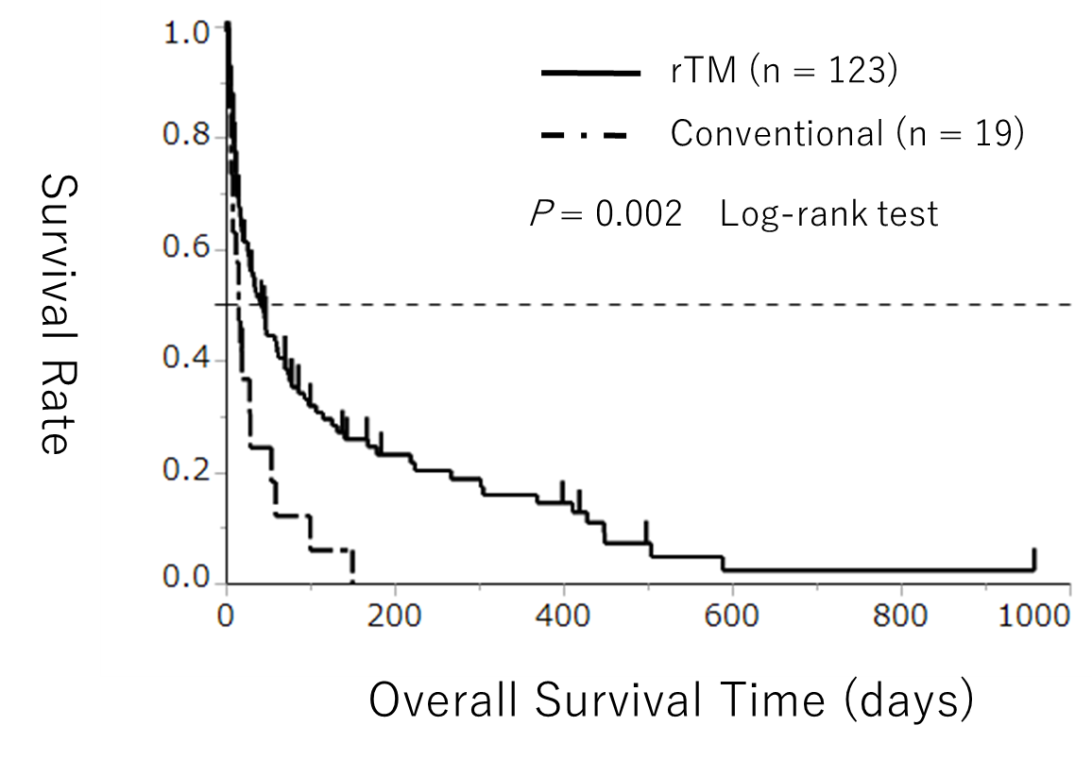
*

Figure legend of Online Resource 2.

Kaplan–Meier survival curves for overall survival duration in the rTM cohort (solid line, n = 123) and conventional treatment cohort (chain line, n = 19).

Online Resource 3

Online Resource 4
